# Review of analytical performance of COVID-19 detection methods

**DOI:** 10.1007/s00216-020-02889-x

**Published:** 2020-09-18

**Authors:** Basant Giri, Shishir Pandey, Retina Shrestha, Krisha Pokharel, Frances S. Ligler, Bhanu B. Neupane

**Affiliations:** 1 Center for Analytical Sciences, Kathmandu Institute of Applied Sciences, Kathmandu, 44600 Nepal; 2 Joint Department of Biomedical Engineering, University of North Carolina at Chapel Hill and North Carolina State University, Raleigh, NC 27695 USA; 3 Central Department of Chemistry, Tribhuvan University, Kathmandu, 44618 Nepal

**Keywords:** Coronavirus, SARS-CoV-2, COVID-19, RT-PCR, Immunoassays, Disease diagnosis

## Abstract

In the recent SARS-CoV-2 pandemic, public health experts have emphasized testing, tracking infected people, and tracing their contacts as an effective strategy to reduce the spread of the virus. Several diagnostic methods are reported for detecting the coronavirus in clinical, research, and public health laboratories. Some tests detect the infection directly by detecting the viral RNA and other tests detect the infection indirectly by detecting the host antibodies. A diagnostic test during the pandemic should help make an appropriate clinical decision in a short period of time. Recently reported diagnostic methods for SARS-CoV-2 have varying throughput, batching capacity, requirement of infrastructure setting, analytical performance, and turnaround times ranging from a few minutes to several hours. These factors should be considered while selecting a reliable and rapid diagnostic method to help make an appropriate decision and prompt public health interventions. This paper reviews recent SARS-CoV-2 diagnostic methods published in journals and reports released by regulatory agencies. We compared the analytical efficiency including limit of detection, sensitivity, specificity, and throughput. In addition, we also looked into ease of use, affordability, and availability of accessories. Finally, we discuss the limitations of the methods and provide our perspectives on priorities for future test development.

## Introduction

The coronavirus disease 2019 (COVID-19) follows multiple past epidemics caused by highly transmissible respiratory viral infections. COVID-19 is caused by the severe acute respiratory syndrome coronavirus 2 (SARS-CoV-2) that was first reported in Wuhan, China [1], and spread into a public health emergency worldwide. Public health experts have emphasized testing as many individuals as possible, tracking infected people, and tracing their contacts as an effective strategy to reduce the spread of the virus. Most of the governments across the globe are exercising this practice to variable extent using an array of testing methods.

The SARS-CoV-2 is a positive-sense single-stranded RNA β family coronavirus that is genetically similar to SARS coronavirus and bat SARS-like coronaviruses [2]. Each virion is 50–200 nm in diameter and consists of four structural proteins named as S (spike), E (envelope), M (membrane), and N (nucleocapsid). The N protein holds the RNA genome of the virus and S, E, and M proteins create the virus envelope together [3]. Recent studies have suggested that bats may be the potential natural host of SARS-CoV-2 [4, 5] and Malayan pangolin the potential intermediate host [6].

Several diagnostic methods have been used to detect the coronavirus in clinical, research, and public health laboratories. Direct tests detect the infection directly by detecting the viral RNA, while indirect tests measure antibodies against the virus in a host that has been exposed. A diagnostic test method should have sufficient sensitivity and accuracy to make appropriate clinical decisions rapidly during a pandemic [7].

**Fig. 1:**
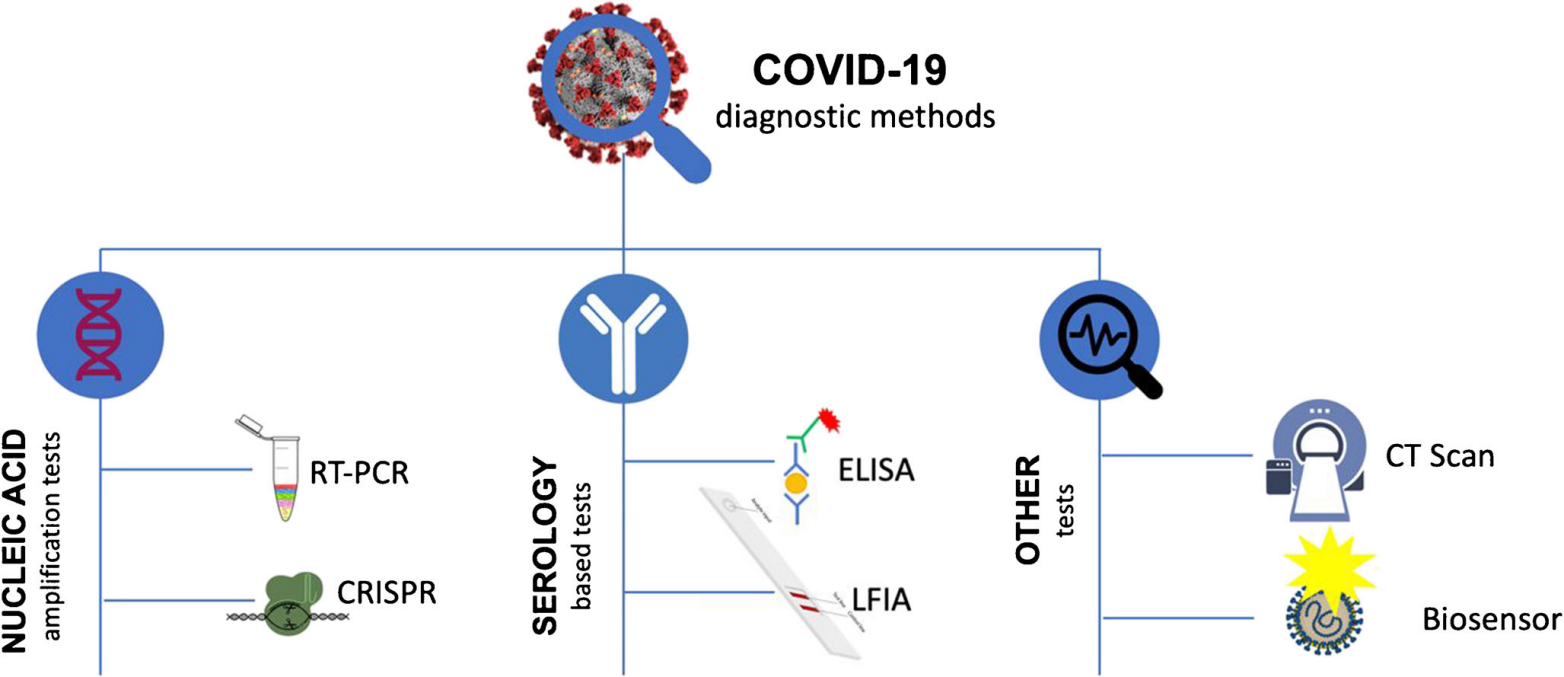
Major diagnostic methods reported for the detection of SARS-CoV-2

Nucleic acid amplification using the reverse transcription polymerase chain reaction (RT-PCR) is the most widely used method for direct SARS-CoV-2 diagnosis [7]. Immunoassays are used to measure the antibodies against the SARS-CoV-2. Emerging methods utilizing CRISPR (clustered regularly interspaced short palindromic repeats) have also been reported, even to the extent of incorporating such tests into nanoparticle-based biosensors. Apart from laboratory-based RT-PCR and immunoassay, several point-of-care (POC) and rapid test methods have become available in the last few months. The major diagnostic methods are illustrated in Fig. 1 and Table 1. Regulatory agencies such as the World Health Organization (WHO) and the US Food and Drug Administration (FDA) have approved the use of a number of diagnostic methods, while some new methods are receiving conditional approval under emergency use authorization (EUA) [22]. These diagnostic methods have varying throughput, batching capacity, requirement of infrastructure setting, analytical performance, and turnaround times [7]. In addition to depending on the equipment and method itself, the result from a method also relies on sample collection protocol, reagents used, potential for cross-contamination, and sample/reagent storage requirements. These factors must be considered while selecting a reliable and rapid diagnostic method to help make an appropriate decision and prompt public health actions.

**Table 1:** Comparison of major diagnostic methods for the detection of SARS-CoV-2

Method	Target analyte	Sample type	LOD	Clinical sensitivity (%)	Specificity (%)	Test time (min)	Advantages	Limitations	References
RT-PCR	Virus mRNA	Respiratory swabs, saliva, sputum, BLF	0.15–100 copy/μL	90–100	100	120–140	High throughput; highly sensitive and specific; detects active cases; useful in clinical decision-making	Labor intensive; requires numerous reagents; specialized equipment; costly; less accurate after ~ 5 days since symptom onset	[8, 9]
CRISPR	Virus mRNA	Respiratory swabs, saliva	2–10 copy/μL	95–100	100	45–70	Simple and efficient; low-cost; low turnaround time; improved specificity; visual readout	Risk of contamination	[10–12]
Molecular POC	Virus mRNA	Respiratory swabs	0.1–10 copy/μL	> 95	100	13–60	Requires low technical manpower; easy to use, faster; cost-efficient; visual readout, community-level testing; may not need RNA extraction; automation	Less accurate after ~ 5 days since symptom onset	[13–15]
NGS	Virus mRNA	Respiratory swabs, BLF	NA	Unbiased	Unbiased	1–2 days	Useful for identifying secondary infections and viral evolution; allows for potential contact tracing	Expensive	[13, 16]
Computed tomography	NA	NA	NA	86–98	25	< 60	Non-invasive, highly sensitive	May not be stand alone	[16, 17]
Biosensors/LFA/RDT	Virus mRNA/antigen/ antibody	Respiratory swabs, blood	0.2 pM	Limited study	Limited study	< 60	Allows real-time detection, faster; easy to use, low-cost	Less accurate	[18, 19]
ELISA	Antibody	Blood	**-**	86–100	89–100	60–180	Useful for disease prognosis and prevalence; needed for identification of convalescent plasma donors	Not suitable for the identification of active cases	[20, 21]

In this paper, we review recently published SARS-CoV-2 diagnosis methods and compare their analytical efficiency in terms of limit of detection (LOD), sensitivity, specificity, and throughput. In addition, we also evaluated ease of use, affordability, and availability of accessories. Note that other tests from a wide variety of companies are being used for COVID-19 diagnostics, with additional products entering the market each week; however, in the rush to get the tests operational, the information on these critical parameters and the company-unique information are often not yet published. Finally, we discuss the limitations of the methods and provide our perspective on priorities for future test development. The intended audience for this paper are students and researchers working on method development and clinical testing of viruses including SARS-CoV-2. People who make decision on which test method should be recommended to a clinical laboratory for the diagnosis of COVID-19 will also benefit from this review paper.

## Sample collection

Two types of sample specimens are being primarily used for the diagnosis of COVID-19. Respiratory specimens are used for direct detection of virus and serum samples are used for identification of antiviral antibodies [23]. Direct detection of viral RNA in wastewater samples is also being used for community surveillance [24]. Similarly, saliva and stool samples have also been explored and require less challenging sampling procedures than respiratory specimens [25]. The respiratory specimens [26] are collected most frequently from the upper respiratory tract (e.g., nasopharynx or oropharynx) and less frequently from the lower respiratory tract (e.g., bronchoalveolar lavage fluid (BLF)). The upper respiratory specimens are collected in the acute phase of infection—ideally within 7 days. Lower respiratory specimens are obtained from patients still symptomatic after more than a week [27, 28]. Apart from nasal and throat specimens, sputum specimens are also collected for the diagnosis of COVID-19 by expectorating deep cough into a sterile container [28]. Serum samples are collected for immunoassay methods. Volume of blood sample for immunoassays ranges from 5 to 10 mL for lab assays to capillary draws of 50–200 μL blood for lateral flow immunoassays (LFIA) [29].

The virus samples should be processed and tested as soon as possible. If immediate testing is not possible, the sample can be stored up to 72 h at 2–8 °C. However, for more than 72 h storage, the specimens should be frozen at -70 °C as soon as possible after collection [28]. It is recommended to avoid repeated freezing and thawing of the specimen [30].

The right type of sample, appropriate collection procedure, and reliable transportation must be in place to minimize the risk of inaccurate results. Based on the stage of infection on a person and the purpose of test, an appropriate sample should be collected. Higher viral loads of SARS-CoV-2 have been reported from specimens collected from nose than from throat [31]. Similarly, higher positive rates were found with nasopharyngeal swabs than oropharyngeal swabs [32]. A case study with a pneumonia patient in Thailand showed a negative test with nasal or oropharyngeal swab samples but a positive test with bronchoalveolar lavage fluid [33]. Sampling both nasal and oropharynx is recommended to minimize the chances of virus detection error [27]. The sensitivity of nasal, nasopharyngeal (NP), and throat swabs was found to be 80%, 90%, and 87%, respectively [34]. The difference in the sensitivity of different types of swabs may depend on disease progression. Therefore, it is important to identify the appropriate type of sample considering the medical condition of the patient and diagnostic facility available for the test. However, further comparisons on the appropriate type of sample needed may be warranted [26, 35, 36].

## RT-PCR tests for virus

The molecular detection methods involve the analysis of nucleic acids present in the sample to identify the virus. The most commonly used laboratory detection method for the clinical diagnosis of COVID-19 is real-time reverse transcriptase polymerase chain reaction (RT-PCR). The same technique has been used in the diagnosis and surveillance of various other viral diseases including SARS-CoV and MERS-CoV [8, 37, 38]. There are a number of commercially available primers and probes used in RT-PCR for the detection of SARS-CoV-2 (see Table 2). Fundamentals of molecular processes for diagnosis of COVID-19 have been covered in a recent review article, and we refer readers to it for more basic information [25].

**Table 2:** List of primers and probes for SARS-CoV-2 [39]

Gene target	Primer/probe	Sequence (5′-3′)	Developed by
RdRp	nCoV_IP2 Fw	ATGAGCTTAGTCCTGTTG	Institut Pasteur
	nCoV_IP2 Rv	CTCCCTTTGTTGTGTTGT	
	IP2 probe	^HEX^AGATGTCTTGTGCTGCCGGTA^BHQ1^	
RdRp	nCoV-IP4 Fw	GGTAACTGGTATGATTTCG	
	nCoV_IP4 Rv	CTGGTCAAGGTTAATATAGG	
	IP4 probe	^FAM^TCATACAAACCACGCCAGG^BHQ1^	
ORF1b	ORF1b-nsp14 F	TGGGGYTTTACRGGTAACCT	Hong Kong University
	ORF 1b-nsp14 R	AACRCGCTTAACAAAGCACTC	
	ORF1b probe	^FAM^TAGTTGTGATGCWATCATGACTAG^TAMRA^	
N	HKU-NF	TAATCAGACAAGGAACTGATTA	
	HKU-NR	CGAAGGTGTGACTTCCATG	
	HKU probe	^FAM^GCAAATTGTGCAATTTGCGG^TAMRA^	
ORF1ab	ORF1ab-F	CCCTGTGGGTTTTACACTTAA	Chinese CDC
	ORF1ab-R	ACGATTGTGCATCAGCTGA	
	ORF1ab probe	^FAM^CCGTCTGCGGTATGTGGAAAGGTTGG^BHQ1^	
N	N-F	GGGGAACTTCTCCTGCTAGAAT	
	N-R	CAGACATTTTGCTCTCAAGCTG	
	N probe	^FAM^TTGCTGCTGCTTGACAGATT^TAMRA^	
N	CDC_N1-F	GACCCCAAAATCAGCGAAT	US CDC
	CDC_N1-R	TCTGGTACTGCAGTTGAATCTG	
	N1 probe	^FAM^ACCCCGCATTACGTTTGGTGGACC^BHQ1^	
N	CDC_N2_F	TTACAAACATTGGCCGCAAA	
	CDC_N2-R	GCGCGACATTCCGAAGAA	
	N2 probe	^FAM^ACAATTTGCCCCCAGCGCTTCAG^BHQ1^	
RdRp	RdRp_F2	GTGARATGGTCATGTGTGGCGG	Charité
	RdRp_R1	CARATGTTAAASACACTATTAGCATA	
	RdRp-P2 probe	^FAM^CAGGTGGAACCTCATCAGGAGATGC^BBQ^	
	RdRp-P1 probe	^FAM^CCAGGTGGWACRTCATCMGGTGATGC^BBQ^	
E	E_Sarbeco_F1	ACAGGTACGTTAATAGTTAATAGCGT	
	E_Sarbeco_R2	ATATTGCAGCAGTACGCACACA	
	E_Probe	^FAM^ACACTAGCCATCCTTACTGCGCTTCG^BHQ1^	
N	WH-NIC N-F	CGTTTGGTGGACCCTCAGAT	MPH, Thailand
	WH-NIC N-R	CCCCACTGCGTTCTCCATT	
	WH-N probe	^FAM^CAACTGGCAGTAACCA^BHQ1^	
N	NIID_N_F2	AAATTTTGGGGACCAGGAAC	NIID, Japan
	NIID_N_R2	TGGCAGCTGTGTAGGTCAAC	
	NIID_N probe	^FAM^ATGTCGCGCATTGGCATGGA^BHQ^	

*MPH* Ministry of Public Health, *NIID* National Institute of Infectious Diseases

The RT-PCR tests take less than an hour to a couple of days to give results, depending on the version of the PCR. The RT-PCR assay can be carried out in one- or two-step approaches. One-step approach is faster in which both RT and DNA polymerase are combined together to carry out their respective reaction in the same reaction tube and is a preferred approach for the detection of SARS-CoV-2 [13]. The two-step approach involves RT of RNA in one tube and subsequent DNA polymerization in a separate reaction tube. Depending on the type of assay format, a single RT-PCR machine can test one to hundreds of samples at one time.

The RT-PCR test result relies on sample collection, primers and probes used, analysis of fluorescence curves, use of suitable controls, and reliability of the temperature control. A negative control is used to check sample cross-contamination, and the positive control is used to assess the chemical integrity of the reagents, primers, and probes. In addition to these controls, the US CDC recommends the use of a human specimen control (HSC) [40] to ensure successful lysis and integrity of extraction reagents and to minimize false negative results by ensuring collection of enough human cellular material [3].

Respiratory specimens may contain different genera of coronaviruses along with other major viral pathogens. In the last six decades, before SARS-CoV-2, the human population was already infected with six other members (229E, OC43, SARS-CoV, NL63, HKU1, and MERS-CoV) of the CoV family [41]. False positive results occurring due to the cross-reactivity with these viruses, human genome, and microflora can be obliterated with the sequence fidelity. In silico analysis using the many sequences available on publicly available databases (e.g., GenBank, the European Molecular Biology Laboratory (EMBL), Global Initiative on Sharing All Influenza Data (GISAID) to discriminate the SARS-COV-2 from other respiratory viruses is a hallmark widely employed to generate a specific primer for COVID-19 detection.

### Laboratory RT-PCR tests

The RT-PCR assays in centralized laboratories are generally performed in 96-well plates for signal reading in batches. The high-throughput 384-well assay system using lower volume was reported recently with detection sensitivity down to 5 copies of viral genome per microliter [42]. The high-throughput method yielded 100% sensitivity and specificity.

The US CDC real-time RT-PCR diagnostic panel under EUA targets two different loci of the N gene [40]. The FDA has already issued several other molecular in vitro diagnostics under EUA [43]. In many protocols, RT-PCR assay of more than one gene target is performed for the positive authenticity of COVID-19. The US CDC considers positive results only when both gene targets (N1 and N2) are positive [40]. If any of the two assays are negative, the result is inconclusive, and the assay has to be repeated following strict guidelines. Positive confirmation with a single gene target is possible if the amplicons are subjected to deep sequence analysis. The protocol from Pasteur Institute [39] utilizes IP2 and IP4 gene targets as the first-line screening tool, while confirmatory testing utilizes the E gene target. The Charité protocol uses the E gene as the screening assay followed by confirmatory assay with the RdRp gene [39]. The Chu et al. protocol recommends the N gene for screening, while ORF1b provides a confirmatory test [44]. A candidate assay targeting RNA sequences coding for the viral E and N proteins and RNA-dependent RNA polymerase (RdRp) showed good alignment of the selected primers and probes with the SARS-CoV-2 genome [8].

Primer sets (IP2 and IP4) designed by Pasteur Institute, when used individually in an assay, can detect about 100 copies of RNA genome equivalent per reaction at 95% detection probability. A lower LOD of 10 copies was obtained with a multiplex assay using these primer sets [39]. Chu et al. used the cloned DNA plasmid containing SARS-CoV ORF1b and N gene to calculate the LOD of their assay. With a known titer of viral RNA, their preliminary study showed the LOD of < 10 copies per reaction [44]. A dynamic range of seven orders of magnitude (2 × 10^−4^ to 2 × 10^3^ TCID_50_/reaction) was obtained using RNA extracted from cells infected by SARS coronavirus as a positive control using one-step RT-PCR assays to detect two different regions (ORF1b and N) of the viral genome [44].

Further improvement in the sensitivity was found in the Charité protocol [8]. They conducted a proficiency testing for the sensitivity with E Sarbeco and RdRp genes using in vitro transcribed RNA derived from SARS-CoV strain Frankfurt-1, where they found LOD of 5.2 and 3.8 copies per reaction, respectively, which was in good agreement with other participating laboratories. They also found the N gene assay was slightly less sensitive than the RdRp and the E Sarbeco gene. The US CDC diagnostic panel showed the LOD of 1 copy of RNA per microliter for both N1 and N2 primer sets [40]. However, sensitivity was affected by the use of different RNA extraction and purification protocols. A comparative study [9] on the analytical sensitivity of four commonly used assays approved by the WHO found the nCoV-N2 primer set from the US CDC was prone to background amplification, which impairs the ability to distinguish between true positive and negative results at low virus concentration. The same study showed primer/probe sets E Sarbeco from the Charité protocol and ORF1b-nsp14 from the Hong Kong University (HKU) protocol were the most sensitive with an LOD of 10 virus genome equivalents per microliter at 75% detection frequency [9].

The PCR assays developed by Pasteur Institute have been claimed to not cross-react with respiratory viruses like influenza A (H1N1, H3N2), enterovirus, adenovirus, human coronaviruses (HKU1, OC43, 22E, NL63), and MERS-CoV, indicating 100% specificity. Assays from HKU are specific to only subgenus Sarbecoviruses [44]. This assay did not distinguish between SARS-CoV and SARS-CoV-2 transcripts because SARS-CoV-2 shares a 79.6% sequence identity with SARS-CoV BJ01 [5], and many of the regions homologous with the primers are conserved nucleotide regions [4]. In contrast, another comparative study showed that the N2 primer/probe set developed by US CDC was highly sensitive comparable with the E Sarbeco set described in Charité assay at low viral copy number [45]. A PCR assay with absolute exclusiveness to SARS-CoV-2 was made possible in the Charité protocol with the inclusion of additional probe (RdRp_P2) (see Table 2) that anneals to only SARS-CoV-2 mRNA transcript. Nonetheless, this protocol does not discriminate between clades of the Sarbecoviruses like SARS-related CoVs from bats. This overlap is corroborated with the sim plot of SARS-CoV-2 showing more than 96% identity to a bat coronavirus [5]. Primers based on the receptor binding domain of the S gene developed by Zhou et al. could discriminate SARS-CoV-2 from bat SARSr-CoV WIV1 [5]. Assays (N1 and N2) based on the US CDC–developed method could also provide specific detection against SARS-CoV-2 [46]. Diagnostic sensitivity and specificity of various methods are given in Table 3.

**Table 3:** Comparison of nucleic acid amplification methods for SARS-CoV-2

Method	Target gene	LOD (copy/μL)	Sensitivity (%)	Specificity	Assay reaction time (min)	Assay sample-to-result time*	Assay results	Reference
CDC qRT-PCR	N1, N2, N3	1^a^	100^b^	100	120	4 h	Quantitative	[40, 45]
DETECTR, RT-LAMP/Cas12	E and N	10	95^c^	100^c^	30–40	45 min	Qualitative	[10]
CRISPR-nCoV	ORF1ab	7.5	100^d^	100^d^	40	< 60 min	Qualitative	[11]
STOPCovid	N	2.2	100^e^	100^e^	60	70 min	Qualitative	[12]
384 RT-PCR method	N1, N2	5	100^f^	100^f^	73.2	NA	Quantitative	[42]
Pasteur Institute	RdRp	4	No data	100	208	NA	Qualitative	[39]
China CDC	ORF1ab, N	10-ORF1ab, 100-N	77^g^	98^g^	31	NA	Qualitative	[9, 39, 47]
NIID, Japan	N	5	No data	100	95	NA	Qualitative	[48]
Charité, Germany	RdRp, E	0.208-E, 0.152-RdRp	100^b^-E	100^b^	46.75	6–7 h	Qualitative	[8, 45, 97]
COVID-19-RdRp/Hel	RdRp/helicase	0.52	43.6^h^	100^h^	41.25	NA	Qualitative	[49]
Exo-IQ-RT-RPA assay	N	1.2	100^i^	100^i^	15–20	< 50 min	Qualitative	[52]
HKU Hong Kong	ORF1b, N	< 0.5	No data	100	75	NA	Qualitative	[44]
NIH, Thailand	N	No data	No data	No data	65.7	NA	Qualitative	[39]
Early-detection RT-LAMP	N	10	100^j^	98.7^j^	30	< 60 min	Qualitative	[53]
RT-RAA	S, ORF1ab	10-S, 1-QRF1ab	100^k^	100^k^	15	< 60 min	Qualitative	[15]
OSN-qRT-PCR	ORF1ab, N	1	100^l^	100^l^	86	2 h	Quantitative	[50]

*including extraction. *Exo-IQ-RT-RPA* exo-internally quenched-reverse transcriptase recombinase polymerase amplification, *OSN* one-step single-tube nested, *RAA* recombinase-aided amplification

^a^LOD determined with QIAGEN QIAmp DSP viral RNA mini kit. ^b^Sensitivity and specificity were calculated from 10 PCR-positive samples (NP/OP) and 297 negative samples respectively. ^c^Sensitivity and specificity were evaluated from 36 PCR-positive and 47 negative NP/OP swabs respectively. ^d^Sensitivity and specificity were calculated from 52 metagenomics NGS-positive specimens (NP/BLF) and 62 negative specimens respectively. ^e^Sensitivity and specificity were based on 12 positive and 5 negative NP specimens respectively. ^f^Sensitivity and specificity were derived from 10 positive and 10 negative NP/OP specimens respectively. ^g^Sensitivity and specificity were constructed based on a Bayesian approach. ^h^Sensitivity and specificity were based on laboratory-confirmed positive respiratory (120) and non-respiratory (153) specimens and 39 negative specimens respectively. ^i^Sensitivity and specificity were derived from 9 PCR-positive and 11 negative samples. ^j^Sensitivity and specificity were deduced from 14 positive nasal swabs and 140 negative respiratory specimens. ^k^Sensitivity and specificity were evaluated from 22 PCR-positive samples and 98 negative NP sputum samples respectively. ^l^Sensitivity and specificity were based on 25 PCR-positive and 142 negative samples respectively

Multiple studies have shown that the clinical sensitivity of a RT-PCR assay is under the influence of specimen type, amount of virus in a swab and the specimen collection time in relation to the onset of symptoms. In one study with 205 patients, RT-PCR sensitivity was 93% for BLF, 72% for sputum, 63% for nasal swabs, and only 32% for throat swabs [23]. The presence of viral load that is below the assay’s LOD will also elicit false negative results. Therefore, a judicious way to increase viral load is to collect combined nose and throat swabs. Viral kinetics of SARS-CoV-2 showed that the viral load in respiratory specimens often peaks in the first week of illness and decreases as the disease progresses [54]. This posited appropriate sample collection times to enhance sensitivity. These findings are similar to a report that showed a 100% positive RT-PCR result by week 1 after onset of symptoms, followed by 89.3%, 66%, and 32% at week 2, week 3, and week4, respectively. By week 5, the positive detection rate plummeted down to only 5.4% [55]. In contrast to the widely used NP swabs, a study more recently showed an increase in the sensitivity by 13% when saliva samples were used [56]. Virus titers from saliva samples were found significantly higher than NP swabs and more importantly, unlike NP swabs, less temporal variation in viral titer was observed with longitudinally collected saliva samples. To accurately estimate diagnostic sensitivity, a clear-cut generalization regarding specimen quality through a rigorous study with large sample size on the dynamic of viral shedding and its correlation across the time course of infection is required.

### POC tests for SARS-CoV-2 genes

The point-of-care (POC) methods are less complex to perform, give results within several minutes, and can be performed on site. They may provide alternative diagnostic methods more suitable for widespread population testing.

A preliminary study reported a POC system capable of detecting genes coding for the N protein of SARS-CoV-2 in respiratory samples [14]. Molecular POC tests utilize RT-PCR or loop-mediated isothermal amplification (LAMP) or other isothermal nucleic acid amplification methodologies [57]. These devices process 1–2 samples at a time. Some of the sample-processing steps can be automated to reduce the test time to under 1 h. While public information on these systems is still limited, the LOD of one such device is reported to be in the range of 120–200 genome equivalents/mL [57].

In an effort to enhance the sensitivity of the LAMP method, a two-stage isothermal amplification (COVID-19 Penn-RAMP) method has been described, producing 10 to 100 fold better sensitivity than conventional LAMP and RT-PCR tests. This test is carried out in a closed tube and employs fluorescence or colorimetric detection. This POC method has been proposed as a rapid, highly sensitive molecular test amenable for use at home, in the clinic and at point of entry by minimally trained individuals and with minimal instrumentation. This method as reported, however, was not yet tested in patient samples [58].

Two of the first POC platforms receiving EUA from FDA are produced by Abbott and Cepheid. The Abbott ID NOW COVID-19 system gives positive results in 5 min and negative results in 13 min after sample preparation. The Xpert® Xpress SARS-CoV-2 system can give results in ~ 30 min with less than a minute sample preparation. It is interesting to note that the Abbott technology missed one-third of samples detected positive by Cepheid Xpert Xpress [59].

The ePlex® SARS-CoV-2 is a qualitative automated nucleic acid multiplex platform and sample-to-answer system capable of automating extraction, amplification, and detection in a single-use cartridge. A simple visual readout similar to a pregnancy test has been described. This POC system is based on SHERLOCK technology and has been called STOPCovid (SHERLOCK Testing in One Pot) [12]. It uses AapCas12b protein coupled with guided RNA that contains spacer sequence for the specific detection of SARS-CoV-2 N gene. It was anticipated that this technique could be deployed at home using saliva samples and cheaply available cookers for LAMP. Some of these EUA technologies may need more rigorous testing to make sure of their reliability and sensitivity.

### Limitations of RT-PCR methods

While having the advantage of accommodating large batches of samples, the conventional RT-PCR test and the facility it requires, including the PCR machines, are too expensive. When transport to a competent laboratory is included, the turnaround time is measured in days. Additionally, sample preparation and assay procedures require well-trained manpower. These shortcomings limit the wider use of the technology during viral pandemics. In addition, high demand during the pandemic creates a shortage of swabs, personal protective equipment, PCR reagents, and equipment such as thermocyclers and biosafety level-2 cabinets, further delaying the diagnosis. Since RT-PCR assays amplify specific target loci, the assays will report a negative result if the particular target locus is not present in the sample. Collective genetic data indicate changes in the viral genome due to insertion or deletion, recombination, and interchange are frequent among CoVs [60, 61]. If this is the case, even a single-nucleotide polymorphism due to mutation at the primer or probe binding site could vitiate the true RT-PCR result. The sensitivity may not be enough to detect early infections due to low concentrations of the virus, especially in asymptomatic cases and may result in false negative results. During the COVID-19 pandemic, there is a growing concern about the inaccessibility of RNA-based testing methods and a global shortage of reagents [62].

### Emerging techniques

Apart from conventional RT-PCR, additional molecular diagnostic tools are emerging for SARS-CoV-2. Whole-genome sequencing of SARS-CoV-2 has the potential to overcome the limitations of RT-PCR. Genomic sequencing can detect fragments even when a complete genome is not present in the sample. Notably, the SARS-CoV-2 genome is free of repeats, making it susceptible to complete characterization using short sequence reads. The genome sequencing involves the construction of RNA library, next-generation sequencing (NGS) of the RNA construct, de novo assembly of the quality-trimmed reads to generate a contig map, and phylogenetic analysis [63]. The genome sequencing may overcome the false results arising from specious priming. However, due to cost, technical complexities associated with the instrument, data analysis, and higher turnaround time of 24 to 48 h, this method may not be a routine use for clinical diagnosis during pandemic [64]. A novel method based on the Sanger sequencing was developed by the Chandler-Brown group that targets the SARS-CoV-2 N protein [65]. This method uses COVID-19 spike-in DNA as an internal control that provides quantification of the viral mRNA. The major advantages of this method are the omission of RNA extraction procedure so there will be no impedance in the testing capacity due to shortage of RNA extraction kits and potential for a very high throughput of one million tests per day with proper customization of the sequencer.

Another emerging method is CRISPR-based technologies. A rapid (< 40 min), easy-to-implement, and accurate CRISPR–Cas12-based lateral flow assay for the detection of SARS-CoV-2 from respiratory swab RNA extracts has been recently reported. The CRISPR-based DETECTR (DNA Endonuclease-Targeted CRISPR Trans Reporter) has a visual read for results. This assay performed simultaneous RT and LAMP steps, followed by Cas12 detection of predefined coronavirus sequences, after which cleavage of a reporter molecule confirms detection of the virus. The LOD of this method was 10 copies per microliter reaction. Clinical sensitivity and specificity were 95% and 100%, respectively, for the detection of the coronavirus in 83 total respiratory swab samples [10]. Another CRISPR-based detection system called SHERLOCK (Specific High Sensitivity Enzyme Reporter UnLOCKing) that targets S and ORF1ab gene fragments of SARS-CoV-2 was described by Sherlock Biosciences. This method was able to detect synthetic COVID-19 RNA sequence in a range between 10 and 100 copies per microliter and could be completed within 1 h [66]. A new platform with engineered complementary recombinant RNA (crRNA) has been reported with LOD as low as ~ 700 fM cDNA from HIV, 290 fM RNA from HCV, and 370 fM cDNA from SARS-CoV-2 in 30 min without a need for target amplification. In this case, the isothermal amplification of SARS-CoV-2 RNA was performed using RT-LAMP, and the modified crRNAs were incorporated in a paper-based lateral flow assay that could detect the target within 40–60 min [67].

Droplet-based digital PCR (dPCR) methods have also been sought as a more sensitive method to test for the RNA of SARS-CoV-2. This method, when tested in 77 patient samples, showed an improved sensitivity from 44 to 94% and the same specificity when compared with the RT-PCR method [68]. The LOD of the same method has been reported to be 2.1 copies/reaction for ORF1ab and 1.8 copies/reaction for N primer/probe set. In a similar dPCR method, the LOD was 2 copies/reaction [69]. When tested in 103 fever-suspected patients, the dPCR method improved the sensitivity from 28.2% with RT-PCR to 87.4% with dPCR.

A dual-functional plasmonic biosensor that combined the plasmonic photothermal effect and localized surface plasmon resonance sensing transduction was reported as an alternative for the clinical diagnosis of COVID-19 [18]. Two-dimensional gold nano-islands functionalized with complementary DNA receptors were used to detect SARS-CoV-2 through nucleic acid hybridization. The biosensor was able to detect 0.22 pM and allowed precise detection of the specific target in a multigene mixture. Gold nanoparticles (AuNPs) functionalized with antisense oligonucleotides were developed for the simultaneous detection of two regions of the SARS-CoV-2 N gene [70]. In the presence of RNA target, AuNPs agglomerate, and the agglomeration is further enhanced by the addition of RNAse H enzyme to generate a distinguishable visible precipitate. An electrochemical ultrasensitive POC device named eCovSens detects the spike protein of SARS-CoV-2 within 10 to 30 s [71]. This device was fabricated by immobilizing antibody against S1 protein on screen-printed carbon electrodes. With spiked saliva samples, the LOD of the device was found to be 90 fM.

Though relatively slow and expensive, computed tomography (CT) of the chest has also been explored in the diagnosis of COVID-19 as a complementary tool to molecular techniques when combined with medical history and clinical observations [65]. In a recent study, artificial intelligence artificial intelligence (AI) algorithms were used to integrate chest CT findings with clinical symptoms, exposure history, and laboratory testing to rapidly diagnose patients who are positive for COVID-19 [51]. The AI system had equal sensitivity as compared with a senior thoracic radiologist. In addition, it improved the detection of patients who were positive for COVID-19 via RT-PCR who presented with normal CT scans, correctly identifying 17 of 25 (68%) patients, whereas radiologists classified all of these patients as negative for COVID-19. When CT scans and associated clinical history are available, the proposed AI system can help to diagnose COVID-19 patients. The supplementary CT scan imaging may help to rule out negative RT-PCR results [16].

## Immunoassays for antibody to virus

Immunoassays are one of the widely used bioanalytical methods to detect and/or quantify an analyte using an antigen-antibody interaction. While there are a few reports using antibodies in a biosensor to detect virus directly [19], the affinity of most antibodies is not sufficient for direct detection of small numbers of virus particles. Most immunoassays used for testing individuals for COVID-19 exposure detect specific antibodies (IgG and IgM) in the serum that react with SARS-CoV-2 proteins. IgM is expressed earlier during an infection (~ 3–6 days) and IgG is the late antibody detectable only after ~ 8 days. IgG is generally more specific for a protein antigen than IgM and affinity generally increases with continued exposure to the (viral) antigen [72].

Enzyme-linked immunosorbent assays (ELISAs) and lateral flow immunoassays (LFIAs) are the most widely practiced techniques to detect the antibodies against SARS-CoV-2 [73]. A two-stage ELISA protocol was described for measuring human antibody responses to the recombinant receptor-binding domain (RBD) of the spike protein or full-length spike protein of SARS-CoV-2 by Krammer’s group from the Icahn School of Medicine in New York [74]. The first stage included a high-throughput screening of samples in a single-serum dilution against the RBD, followed by a second stage in which positive samples from the first stage underwent a confirmatory ELISA against the full-length spike protein.

Recently, a peptide-based magnetic chemiluminescence enzyme immunoassay was developed for the diagnosis of COVID-19 [75]. The test has high throughput and can perform simultaneous clinical tests for other biomarkers, such as C-reactive protein (CRP), which should also be tracked in COVID-19 suspects [76].

The sensitivity of the immunoassays for the presence of antibodies in human samples may depend on the viral titer and time of sample collected after viral infection; both factors impact circulating antibody concentration [77]. ELISA and chemiluminescence assays based on the antibodies have shown a sensitivity of 70–95% and 82–97% respectively [78–80]. A peptide luminescence method was developed with recombinant S protein to detect IgM and IgG antibody. The total positive rate of detection was 81% with < 6% coefficient of variation.

The immunoassay-based rapid diagnostic test (RDT) kits, depending on the vendor type, are reported to have sensitivity in the range of 60–80% and selectivity of 85–100% at a confidence interval of 95% [57]. The RDTs that are LFIAs have shown 86% to 88% sensitivity and 90% to 99% specificity to detect total antibodies [78, 79, 81, 82]. A study by Bendavid et al. demonstrated 82% sensitivity and 99.5% specificity for detecting antibodies to SARS-CoV-2 tested in 3330 adults and children in Santa Clara County, CA, using LFIAs [29]. A test that detects both IgM and IgG produced a sensitivity, specificity, and accuracy of 57%, 100%, and 69% for IgM and 81%, 100%, and 86% for IgG, respectively. Combining the results from both IgM and IgG yielded a clinical sensitivity of 82% [83]. Analytical performance of various immunoassay methods for COVID-19 detection has been summarized in Table 4. A rapid (10 min) and sensitive LFIA that uses lanthanide-doped polystyrene nanoparticles to detect anti-SARS-CoV-2 IgG has also been reported [84].

**Table 4:** Comparison of analytical performances of immunoassay-based COVID-19 diagnostic methods

Manufacturer	Method	Clinical sensitivity (%)	Specificity (%)	Assay time (min)	References
Premier Biotech, Minneapolis, MN	LFIA	80.3	99.5	12–20	[28]
Zhu Hai Liv Zon Diagnostics Inc., China	LFIA	82.4	100	10–15	[79]
AutoBio Diagnostics, Zhengzhou, China	LFIA	93	100	15–20	[21]
Artron Laboratories	LFIA	83	100	15–20	[21]
Shanghai KinBio Inc.	LFIA	88.7	90.6	15	[78]
Epitope diagnostics (EDI), USA	ELISA	100	88.7	80	[20]
Euroimmun, Germany	ELISA	86.4	96.2	60–120	[20]
Zhu Hai Liv Zon Diagnostics Inc., China	ELISA	87.3	100	180	[79]
Mikrogen, Germany	ELISA	86.4	100	120–180	[20]
Shenzhen YHLO Biotech Co., Ltd.	Chemiluminescence	88.9	90	30	[82]
Kangrun Biotech, Guangzhou, China	Chemiluminescence	96.8	99.8	30–35	[83]

The Single Molecule Array (SIMOA) multiplexed method developed by Quanterix targets three immunoglobulin responses (IgG, IgM, and IgA) to four viral proteins (spike protein, S1 subunit, receptor binding domain, and nucleocapsid) in a single sample, thereby enabling the quantification of 12 antibody isotype-viral protein interactions. It gives a high-resolution profile of immune response of SARS-CoV-19 when compared with a traditional ELISA where only a single interaction can be interrogated. The SIMOA method demonstrated a sensitivity of 86% and a specificity of 100% during the first week of infection, and 100% sensitivity and specificity thereafter [85].

RDTs employing serological immunoassays are less complex, cost less than the molecular tests, and can give results in a short time period. Immunoassays are good tools to track and study past infections, especially in asymptomatic cases. Serological assays can be used to determine the infection rate and to estimate the population extent and prevalence of infection. Results from a serological survey can also be used to project mortality rates in a community. Furthermore, they are useful to characterize the immune response to the virus. A serological assay is critical for identifying potential plasma donors and for developing vaccines. These tests are relatively quick, are easy to use, and require no highly trained personnel and sophisticated equipment. By early August 2020, a quick scan of the internet showed over 20 rapid tests under commercialization with the European CE Mark approval.

### Limitations of immunoassay methods

Immunoassays are not as specific as the tests recognizing RNA sequences in the virus. In the past, molecules like interferon, rheumatoid factor, and non-specific IgM have been shown to cause problems in immunoassays [80], and levels of such potential interferents can be highly variable in COVID-19 patients.

Immunoassays to detect the antibody response to virus may produce false negative results during the early stage of the infection. The sensitivity may be low when tested in local populations with asymptomatic or mildly symptomatic individuals who may generate only low-titer antibodies. Sensitivity may be even lower if there are many such cases. Results may also be biased due to prior COVID-like illnesses that confound the specificity of the antibody response. One of the difficulties in validating an assay for antiviral antibodies is the availability of appropriate negative and positive controls. Negative controls are easier to come by and can be serum pools taken before 2019. Positive controls can be convalescent samples from COVID-19 patients or monoclonal antibodies like CR3022 [29]. Despite high sensitivity, the antigen used in an assay might not be an ideal protein of choice to target for diagnostics because the protein is highly conserved across a broad spectrum of coronavirus in many animals, e.g., the N protein of SARS-CoV from feline infectious peritonitis virus and porcine transmissible gastroenteritis virus [78]. In some instances, N protein–based immunoassays were unable to differentiate SARS patients from healthy individuals. So, the serum diagnostics are complex, requiring more than just one antigenic protein to be used as a target [86].

To add to the complexity of interpreting results from diagnostics for antiviral antibodies, the antibody response in a patient depends on age, nutritional status, and existing medical conditions and medications [54, 87]. The majority of patients develop antibodies only after the second week of infection, i.e., in the recovery phase of COVID [88, 89]. By this phase, many of the opportunities for disease intervention are already passed. Antibody tests may also cross-react with other pathogens and human coronavirus and give false positive results [54, 90]. Serological tests may be useful for epidemiology, but not sufficiently reliable for clinical diagnosis [91].

It has been suggested to detect multiple antibodies to avoid false results. Antisera raised against N proteins lack specificity and need to be used in combination with antibodies to other proteins; e.g., antisera against N proteins and S proteins might be used to develop a more reliable serum-based quick diagnostic test kits [80, 92, 93]. To improve the sensitivity, it is paramount to include other biomarkers of the early stage of SARS-CoV-2 infection [76]. The WHO has not recommended the use of antibody-based POC systems in clinical decision-making [18].

## Future perspectives

Among various methods available for the detection of COVID-19, the RT-PCR is the most reliable and widely used method. During pandemic emergencies, shortage of resources such as PCR kits is common. It is therefore important to have multiple options for the diagnostic methods. Alternative testing platforms and accessories that could be locally manufactured, even in a small scale, are equally important. Such platforms would be appropriate in resource-limited settings as well. The currently practiced RT-PCR methods are costly, and therefore many countries, especially the low-income ones, cannot afford enough number of COVID-19 tests to screen larger population. Important gaps remain in screening asymptomatic persons in the incubation phase. Accurate determination of live viral shedding among patients in the convalescence phase to inform de-isolation decisions is also challenging.

Quality assurance and regulatory frameworks surrounding testing remain a challenge. There are several instances of COVID-19 test kits being recalled in several countries due to suspicious quality. The lack of an established reference standard, the use of differing sample collection and preparation methods, and an incomplete understanding of viral dynamics across the time course of infection hamper the rigorous assessment of the diagnostic accuracy of the many newly introduced SARS-CoV-2 assays [7].

New technologies such as CRISPR-based diagnostics and implementation of practical approaches for POC applications such as RT-LAMP will provide rapid, simple, low-cost, portable, temperature-stable assay systems that are appropriate resource-limited settings, not only for doctor’s offices but also for airports, border crossings, and remote locations. One of the research areas to be explored for diagnosis of COVID-19 is to perform the serological assays in microfluidic POC systems. Microfluidic systems offer advantages of small sample volume, miniaturization, portability, multiplexed analyses, and rapid detection and can increase the sensitivity of analyte detection using signal amplification strategies [94]. The application of such a system for the diagnosis of HIV patients was previously reported with sensitivity, specificity, and test time of 92–100%, 79–100%, and 15 min, respectively [95]. Simple antigen-based immunoassay POC tests and molecular POC system based on paper microfluidics are also promising alternatives. A LAMP-on-paper POC system has been reported for the detection of dengue virus [95]. A conceptual framework of such a system for COVID diagnosis has already been provided by Yang et al. [96]. The integration of smartphones, which are common in most parts of the world, with the POC diagnostic system is promising to read, analyze, and report the assay results. Considering the fact that the possibility of COVID-19 infection will remain in the population like the flu viruses, a multiplex testing method for multiple diseases should be considered as a routine testing platform in future.
